# Computational modeling and characterization methods for rotating magnetic nanochain-enhanced lateral flow immunoassays^☆^^☆☆^

**DOI:** 10.1016/j.mex.2026.103936

**Published:** 2026-05-05

**Authors:** Alexey V. Orlov, Juri A. Malkerov, Alexandra S. Rakitina, Anastasiia Kudriavtseva, Alexander A. Minakov, Daniil I. Tselikov, Petr I. Nikitin, Slavko Kralj

**Affiliations:** a Prokhorov General Physics Institute of the Russian Academy of Sciences, Moscow, Russia; b Central University, 7 Gasheka St., 123056 Moscow, Russia; c National Research Nuclear University MEPhI (Moscow Engineering Physics Institute), 31 Kashirskoe shosse, Moscow 115409, Russia; d Moscow Center for Advanced Studies, 20 Kulakova Str., Moscow, Russia; e Jožef Stefan Institute, Department for Materials Synthesis, Jamova cesta 39, Ljubljana, Europe SI-1000, Slovenia; f University of Ljubljana, Faculty of Pharmacy, Aškerčeva 7, Ljubljana, Europe SI-1000, Slovenia

**Keywords:** Lateral flow immunoassay, Magnetic nanochains, Computational fluid dynamics, Rotating magnetic field, Point-of-care diagnostics, Cardiac biomarkers

## Abstract

This article provides comprehensive methodological guidance for implementing rotating magnetic nanochain-enhanced lateral flow immunoassays with volumetric magnetic detection. Rotating magnetic nanochains act as microscale stirrers that substantially enhance antibody-antigen binding kinetics through convective mixing, yet their integration into lateral flow platforms presents unique technical challenges requiring both computational optimization and specialized characterization. We describe complete workflows for: (i) computational fluid dynamics modeling using COMSOL Multiphysics to simulate nanochain rotation, fluid flow, and mass transport enhancement; (ii) electron microscopy characterization of magnetic nanochain morphology and size distributions; (iii) rotating magnetic field generator design and operation; and (iv) magnetic particle quantification measurement procedures for volumetric signal readout. Each section provides step-by-step instructions with sufficient detail to enable independent replication. The described methods enable development of lateral flow assays achieving sub-nanogram detection limits with rapid (6-minute) analysis times, addressing critical needs in point-of-care diagnostics. These methods complement our related research article in Biosensors and Bioelectronics by providing the technical foundation necessary for adoption and adaptation of this technology by other laboratories.

• COMSOL Multiphysics workflow for modeling convective enhancement by rotating magnetic nanochains
• Electron microscopy procedures for comprehensive nanochain characterization
• Instrumentation methods for rotating field generation and volumetric magnetic particle quantification

## Graphical abstract

**Figure:**
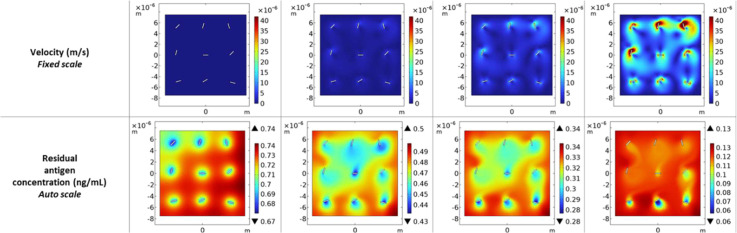

**Specifications table**

**Table:** 

**Subject area**	Chemistry
**More specific subject area**	Biosensor development, computational fluid dynamics modeling, and magnetic nanoparticle characterization for point-of-care diagnostics
**Name of your method**	Computational modeling and characterization of rotating magnetic nanochain-enhanced lateral flow immunoassays
**Name and reference of original method**	N/A
**Resource availability**	N/A

## Background

Lateral flow immunoassays represent established point-of-care diagnostic platforms valued for simplicity, rapid results, and minimal equipment requirements. However, their analytical sensitivity often remains insufficient for detecting low-abundance biomarkers at clinically relevant concentrations, particularly for applications requiring sub-nanogram-per-milliliter detection limits. Traditional enhancement strategies—including signal amplification, engineered nanolabels, and sensitive optical readout—address detection sensitivity but do not fundamentally improve the underlying immunoreaction kinetics that limit assay performance in rapid formats.

Recent advances have demonstrated that rotating magnetic nanochains can substantially enhance immunoassay kinetics through microscale convective mixing. When subjected to rotating magnetic fields, anisotropic magnetic nanostructures act as nanoscale stirrers, generating local fluid flow that reduces diffusion limitations and accelerates antibody-antigen binding. This concept has been successfully demonstrated in controlled microplate-based immunoassays. However, translating this approach to lateral flow platforms presents distinct technical challenges: the porous membrane architecture, unidirectional capillary-driven flow, open geometry, and requirement for portable instrumentation create conditions fundamentally different from static microplate wells.

Successful implementation of rotating nanochain-enhanced lateral flow immunoassays requires integration of multiple technical components: understanding fluid dynamics through computational modeling, characterizing nanochain physical properties, generating appropriate rotating magnetic fields, and detecting accumulated magnetic labels. Each component demands specialized expertise and careful optimization. Furthermore, the interplay between magnetic rotation and capillary flow—two simultaneous transport phenomena—creates complexity not easily addressed through empirical optimization alone.

This methodological article provides comprehensive technical guidance enabling independent laboratories to implement and adapt rotating magnetic nanochain-enhanced lateral flow immunoassays. We focus on four critical technical areas: (i) computational fluid dynamics modeling to understand and optimize convective enhancement mechanisms; (ii) electron microscopy characterization to verify nanochain morphology and size distributions; (iii) rotating magnetic field generation using practical, reproducible instrumentation; and (iv) volumetric magnetic particle quantification for sensitive, quantitative readout.

Computational modeling forms a particularly critical component. Understanding how rotation frequency, field amplitude, nanochain geometry, and flow conditions affect convective enhancement requires numerical simulation. However, modeling rotating anisotropic particles in flowing media through porous structures presents significant computational challenges. We provide complete workflows in COMSOL Multiphysics—from geometry construction through validation—that enable researchers to model their specific configurations, predict performance, and optimize parameters computationally before extensive experimental work.

This methods article complements our related research publication in Biosensors and Bioelectronics [1], where we report analytical performance, clinical validation, and scientific insights. Specifically, analytical validation using clinical matrices — including spike-recovery experiments in pooled human serum and specificity testing against a panel of six clinically relevant biomarkers (AFP, CA125, CEA, cTnI, fPSA, NT-proBNP) — is described in the companion article. While that article presents discoveries and their implications, the current work focuses exclusively on technical reproducibility. Our goal is to transform the rotating nanochain-enhanced lateral flow approach from a demonstrated concept into a reproducible technology accessible to laboratories with appropriate computational and experimental capabilities.

The described methods are particularly relevant for researchers developing ultrasensitive point-of-care diagnostics for low-abundance biomarkers, including cardiac markers, inflammatory mediators, and early-stage disease indicators. The computational procedures are generalizable to other magnetic particle-enhanced assays, while the characterization and instrumentation methods provide practical guidance applicable across magnetic biosensor platforms.

## Method details

The parameter values tabulated throughout this section are specific to the H-FABP assay described in the companion article, employing NC140 nitrocellulose membranes, ∼1 μm magnetic nanochains, and a 15 kDa protein analyte. Researchers adapting these workflows to other systems should recalculate key parameters — in particular, the analyte diffusion coefficient (via Stokes-Einstein, scaled by molecular weight), nanochain dimensions, membrane permeability and porosity (from manufacturer data), and binding kinetic constants (from independent measurements). The COMSOL workflow structure and physics coupling remain generally applicable.

## Antigen capture efficiency measurement procedure

This procedure enables direct quantification of antigen binding to magnetic nanochains under different conditions (e.g., with or without magnetic rotation).

*Materials and equipment*

• Functionalized magnetic nanochains
• Target antigen (H-FABP or other)
• Running buffer: PBS pH 7.4 containing 10 % (v/v) antigen-free pooled human serum, 1 % (w/v) BSA, and 0.1 % (v/v) Tween-20
• Permanent magnet (neodymium, N52, ≥0.3 T surface field)
• Micropipettes (10–100 µL)
• Microcentrifuge tubes (1.5 mL)
• Lateral flow test strips for quantification

*Sample preparation:*

• Prepare antigen solution at known concentration (e.g., 1.0 ng/mL) in running buffer
• Aliquot 50 µL into microcentrifuge tubes
• Prepare at least triplicate samples for each condition

*Nanochain incubation:*

• Add predetermined amount of functionalized nanochains (e.g., 3 µg) to each sample
• For rotation condition: apply rotating magnetic field (optimized parameters: 3 Hz, 200 Oe)
• For static condition: incubate without magnetic field
• Control: sample without nanochains
• Incubate for defined time (e.g., 6 min) at room temperature

*Magnetic separation:*

• Place samples on strong permanent magnet
• Allow 2 min for complete sedimentation of magnetic nanochains
• Nanochains will form visible pellet on tube wall adjacent to magnet
• Supernatant collection:
• Carefully pipette supernatant (40 µL) without disturbing nanochain pellet
• Transfer to clean tube
• Dilute as needed for measurement range
• MPQ reader (or alternative quantitative readout)

*Residual antigen quantification:*

• Measure residual antigen concentration using lateral flow assay (without rotation) or other quantitative method
• Use calibration curve prepared from known antigen concentrations
• Perform all measurements in triplicate

### Data analysis

• Calculate capture efficiency:

*Capture efficiency (* *%) = [(C₀ - Cₛ) / C₀] × 100,* where C₀ = initial concentration, Cₛ = supernatant concentration

• Calculate fold improvement:

*Fold improvement = (Capture efficiency with rotation) / (Capture efficiency without rotation)*

## Rotating magnetic field generator

The mechanical configuration consists of a U-shaped aluminium frame (arm length 120 mm, internal gap 95 mm) mounted on a vertical rotation axis. Two NdFeB permanent magnets (N52 grade, 7.5 × 15 × 80 mm) are positioned symmetrically at adjustable separations (95–185 mm), enabling field amplitude tuning from 70 to 300 Oe at the geometric center. Field amplitude and homogeneity were characterized using a calibrated Hall magnetometer (GS-100DAH, DZJLUCK, China) by mapping at 1 mm intervals across a 10 × 10 mm grid centered on the strip position. Spatial field variations within the central zone remained below 3 %, consistent with finite-element simulations (companion article, Supplementary Note 1, Fig. S1). Rotation frequency was set by DC gear-motor voltage control and verified by optical tachometry. The operating condition of 3 Hz / 200 Oe was selected through the systematic optimization of field amplitude and frequency described in the companion article (Fig. 4a and b).

## Computational fluid dynamics modeling of rotating nanochains

### Software and system requirements

• COMSOL Multiphysics version 5.5 or higher

Required modules:

  CFD Module (for Single-Phase Flow physics)
  Chemical Reaction Engineering Module (for Transport of Diluted Species)

### Model overview and physics

This simulation models the convective enhancement of antibody-antigen binding around rotating magnetic nanochains. The model couples two physics:

• Laminar Flow (Single-Phase Flow, spf): Describes fluid motion induced by nanochain rotation
• Transport of Diluted Species (tds): Describes antigen diffusion, convection, and surface binding

The key mechanism is that rotating nanochains (modeled as rigid rectangular objects with rotating walls) generate local fluid velocity fields that enhance mass transport of antigen molecules toward antibody-functionalized surfaces, where binding occurs according to Langmuir-type kinetics.

### Step-by-step modeling procedure

Step 1: Create new model

• Launch COMSOL Multiphysics
• Model Wizard:
  Space dimension: 2D
• Physics:
  Add Fluid Flow → Single-Phase Flow → Laminar Flow (spf)
  Add Chemical Species Transport → Transport of Diluted Species (tds)

Study: Time Dependent

Step 2: Define global parameters

• Navigate to Global Definitions → Parameters
• Create a parameter node named "Parameters 1″ and define the parameters as listed in Table 1.

**Table 1:** List of global parameters for computational fluid dynamics modeling of rotating nanochains.

**Name**	**Expression**	**Value**	**Description**
L_chain	1050e-9[m]	1.05 × 10⁻⁶ m	Nanochain length
W_chain	150e-9[m]	1.5 × 10⁻⁷ m	Nanochain width
domain_size	15e-6[m]	1.5 × 10⁻⁵ m	Computational domain size
C_bulk	6.67e-8[mol/m^3]	6.67 × 10⁻⁸ mol/m³	Initial antigen concentration (1 ng/mL for 15 kDa protein)
mu_fluid	1.8e-3[Pa*s]	0.0018 Pa·s	Dynamic viscosity of water
rho_fluid	1000[kg/m^3]	1000 kg/m³	Density of water
k_on	1[m^3/(mol*s)]	1 m³/(mol·s)	Association rate constant
k_off	10E-2[1/s]	0.1 s⁻¹	Dissociation rate constant
freq	0[Hz]	0 Hz	Rotation frequency (sweep parameter)
omega	2*pi*freq	0 Hz	Angular velocity (rad/s)
Gamma_max	1e-8[mol/m^2]	1 × 10⁻⁸ mol/m²	Maximum surface binding capacity
t_end	360[s]	360 s	Simulation end time
x_center	0[m]	0 m	Domain center x-coordinate
y_center	0[m]	0 m	Domain center y-coordinate
spacing	domain_size/3	5 × 10⁻⁶ m	Spacing between nanochains
D_analyte	1.1e-10[m^2/s]	1.1 × 10⁻¹⁰ m²/s	Analyte diffusion coefficient (Stokes-Einstein, 15 kDa)

• Define the parameters of nanochain positions (3 × 3 grid) as listed in Table 2

**Table 2:** List of parameters for of nanochain positions.

**Name**	**Expression**	**Description**
x_c1	-spacing	Chain 1, x position
y_c1	-spacing	Chain 1, y position
x_c2	0	Chain 2, x position
y_c2	-spacing	Chain 2, y position
x_c3	spacing	Chain 3, x position
y_c3	-spacing	Chain 3, y position
x_c4	-spacing	Chain 4, x position
y_c4	0	Chain 4, y position
x_c5	0	Chain 5, x position
y_c5	0	Chain 5, y position
x_c6	spacing	Chain 6, x position
y_c6	0	Chain 6, y position
x_c7	-spacing	Chain 7, x position
y_c7	spacing	Chain 7, y position
x_c8	0	Chain 8, x position
y_c8	spacing	Chain 8, y position
x_c9	spacing	Chain 9, x position
y_c9	spacing	Chain 9, y position

Step 3: Define variables for rotation

• Navigate to Definitions → Variables
• Create "Variables 1″ listed in Table 3 with Geometric Entity Selection: Entire model

**Table 3:** List of variables that define the rotational velocity field for each nanochain around its center.

**Name**	**Expression**	**Unit**	**Description**
x_local	x - x_center	m	Local x-coordinate
y_local	y - y_center	m	Local y-coordinate
r_local	sqrt(x_local^2 + *y*_local^2)	m	Radial distance from center
v_tang	omega*r_local	m/s	Tangential velocity magnitude
u_rot	-omega*y_local	m/s	Rotation velocity, x-component
v_rot	omega*x_local	m/s	Rotation velocity, y-component

Step 4: Build geometry

• Navigate to Geometry 1
  Create computational domain
  Right-click Geometry 1 → Rectangle
    ▪ Label: Domain
    ▪ Width: domain_size
    ▪ Height: domain_size
    ▪ Position, Base: Corner
    ▪ x: -domain_size/2
    ▪ y: -domain_size/2
  Click Build Selected
• Create nanochains (repeat for all 9):
  For each nanochain (*i* = 1 to 9):
  Right-click Geometry 1 → Rectangle
    ▪ Label: Chain_i (e.g., Chain_1, Chain_2, …)
    ▪ Width: L_chain
    ▪ Height: W_chain
    ▪ Position, Base: Corner
    ▪ x: x_ci - L_chain/2 (e.g., x_c1 - L_chain/2)
    ▪ y: y_ci - W_chain/2
    ▪ Rotation Angle: Varies by chain (random orientations simulate realistic nanochain configurations in solution.)
  Click Build Selected
• Create fluid domain (domain minus nanochains):
  Right-click Geometry 1 → Boolean Operations → Difference
    ▪ Label: Difference 1
    ▪ Objects to add: Domain
    ▪ Objects to subtract: Chain_1, Chain_2, …, Chain_9 (select all 9)
    ▪ Click Build Selected
  Right-click Geometry 1 → Form Union
  Click Build All Objects

Step 5: Configure Laminar Flow physics (spf)

• Navigate to Laminar Flow (spf)
• Click Fluid Properties 1
  Domain Selection: All domains (domain 1, the fluid region)
• Fluid properties:
  Dynamic viscosity (μ): mu_fluid
  Density (ρ): rho_fluid
• Click Initial Values 1
  Domain Selection: All domains
• Velocity field:
  u: 0
  v: 0
  Pressure: 0
• Right-click Laminar Flow (spf) → Wall
  Label: Wall 1
  Boundary Selection: Manual → Select boundaries of the outer domain (boundary 1–3, or use "All boundaries" then manually deselect nanochain boundaries)
  Boundary condition: No slip
• For each nanochain surface (*i* = 1 to 9): Right-click Laminar Flow (spf) → Wall
  Label: Chain_i_Wall (e.g., Chain_1_Wall)
  Boundary Selection: Manual → Select the 4 boundaries of Chain_i
  Boundary condition: No slip
  Wall Movement:
    ▪ Translational velocity: Manual
    ▪ Velocity of moving wall:
    ▪ u_tr (x-component): -omega*(y - y_ci) (e.g., -omega*(y - y_c1))
    ▪ v_tr (y-component): omega*(x - x_ci) (e.g., omega*(x - x_c1))
• Right-click Laminar Flow (spf) → Pressure Point Constraint
  Point Selection: choose any point in fluid domain (e.g., origin)
    ▪ Pressure: 0

Step 6: Configure Transport of Diluted Species physics (tds)

• Navigate to Transport of Diluted Species (tds)
• Click Transport Properties 1
  Domain Selection: All domains
  Diffusion coefficient: D_analyte (see Table 1)
  Convection:
    ▪ Velocity field: Velocity field (spf) (couples with fluid flow)
• Click Initial Values 1
  Domain Selection: All domains
  Concentration:
    ▪ c: C_bulk
• Right-click Transport of Diluted Species (tds) → No Flux
  Label: No Flux 1
  Boundary Selection: Outer domain boundaries (boundary 1–3)
• Right-click Transport of Diluted Species (tds) → Flux
  Label: Surface Binding
  Boundary Selection: Manual → Select boundaries 4–9 (all nanochain surfaces)
  Flux type: General inward flux
  Species c:
    ▪ Inward flux (J₀,c): -k_on*c*Gamma_max*(1 - c/(k_off/k_on))
• Right-click Transport of Diluted Species (tds) → No Flux
  Label: No Flux 2
  Boundary Selection: any boundaries not covered above

Step 7: Generate mesh

• Navigate to Mesh 1
• Click Size
  Geometric entity level: Entire geometry
  Element size: Predefined → Finer
  Calibrate for: General physics
• Right-click Mesh 1 → Boundary Layers
  Label: Boundary Layers 1
  Boundary Selection: Manual → Select boundaries 4–9 (all nanochain surfaces)
    ▪ Number of layers: 6
    ▪ Stretching factor: 1.2
    ▪ Thickness specification: First layer
    ▪ Thickness: 1e-9 (1 nm)
• Click Build All
• Inspect mesh in Graphics window

Step 8: Configure study (time-dependent with parametric sweep)

• Navigate to Study 1
  Click Step 1: Time Dependent
  Times: range(0, 10, t_end) → simulates from 0 to 360 s with output every 10 s
• Right-click Study 1 → Parametric Sweep
• Move Parametric Sweep node above “Step 1: Time Dependent” in tree
• Click Parametric Sweep
  Sweep type: Specified combinations
  Parameter names: freq
  Parameter value list: range(0,1,20) → [0, 1, 2, 3, …, 20] Hz
  Parameter unit: Hz
• Expand Study 1 → Solver Configurations → Solver 1
• Click Time-Dependent Solver 1
  Time stepping: Free (default, automatic)
  Relative tolerance: 1e-3 (default)
  Absolute tolerance: 1e-6 (default)
• In Parametric Sweep node:
  Keep solutions: All (to enable post-processing for all frequencies)
  Output While Solving: ✓ (optional, for monitoring)

Step 9: Run simulation

• Click Compute button (=) in toolbar
  Simulation will run 21 frequency points
• Monitor progress in Progress window

Step 10: Post-processing - Velocity and concentration fields

• Right-click Results → 2D Plot Group
• Label: Velocity (spf)
• In 2D Plot Group, right-click → Surface
  Expression: spf.U (velocity magnitude)
  Coloring and Style: Jet color table
• Right-click → Arrow Surface
  x-component: u
  y-component: v
  Arrow length: Logarithmic
  Scale factor: adjust for visibility
• Click Plot
• Right-click Results → 2D Plot Group
• Label: Concentration (tds)
• In 2D Plot Group, right-click → Surface
  Expression: c (species concentration, mol/m³)
  Unit: convert to ng/mL: c*Mr/(1e-6) → gives ng/mL
  Coloring and Style: Thermal or Rainbow color table
• Click Plot
• In Velocity (spf) or Concentration (tds) plot group
• Click Plot → Select Parameter selection (freq): All
• Click Play button

Step 11: Quantitative analysis - Calculate binding efficiency

• Right-click Derived Values → Integration → Surface Integration
  Label: Surface Integration 1
  Dataset: Study 1/Parametric Solutions 1 (sol2)
  Parameter selection (freq): All
  Time selection: Last (*t* = 360 s)
  Selection: Domain (entire fluid domain, selection 1)
• In Surface Integration 1, expand Expressions section, add expressions listed in Table 4.

**Table 4:** Expressions to calculate binding efficiency.

**Expression**	**Unit**	**Description**
max(c/((domain_size)^2)*15,000[g/mol], 0)	ng/ml	Residual concentration
C_bulk/((domain_size)^2)*15,000[g/mol]	ng/ml	Initial concentration
(C_bulk - c)/(C_bulk*((domain_size)^2))	1	Binding efficiency

• Click Evaluate
• Right-click Results → 1D Plot Group
  Label: Efficiency vs Frequency
  Right-click → Table Graph
  Table: Select Surface Integration 1 table
  x-axis data: freq column
  y-axis data: Efficiency column
• Click Plot

## Computational modeling of nanochain transport and cooperative binding in porous lateral flow membrane

### Software and system requirements

• COMSOL Multiphysics version 5.5 or higher
• Required modules:
  CFD Module (for Darcy's Law physics)
  Chemical Reaction Engineering Module (for Transport of Diluted Species)

### Model overview and physics

This simulation models nanochain transport through a porous nitrocellulose membrane with capillary-driven flow and surface binding at the test line. The model couples three physics:

• Darcy's Law (dl): Describes fluid flow through porous medium driven by capillary pressure
• Transport of Diluted Species - Nanochains (tds): Describes convection and diffusion of free nanochains
• Transport of Diluted Species - Antigen-Antibody Complexes (tds2): Describes free antigen and bound antibody-antigen complexes

### Step-by-step modeling procedure

Step 1: Create new model

• Launch COMSOL Multiphysics
• Model Wizard:
  Space dimension: 2D
• Physics:
  Add Fluid Flow → Porous Media and Subsurface Flow → Darcy's Law (dl)
  Add Chemical Species Transport → Transport of Diluted Species (tds) [for nanochains]
  Add Chemical Species Transport → Transport of Diluted Species (tds2) [for antigen-antibody]

Step 2: Define global parameters

• Navigate to Global Definitions → Parameters
• Create parameter node "Parameters 1″ and define parameters as listed in Table 5.

**Table 5:** Global parameters for Darcy flow model.

**Name**	**Expression**	**Value**	**Description**
L_model	3 [mm]	0.003 m	Membrane length
H_membrane	2.5[mm]	0.0025 m	Membrane thickness
porosity	0.75	0.75	Porosity
k_perm	1e-13[m^2]	1 × 10⁻¹³ m²	Permeability
pore_size	20[um]	2 × 10⁻⁵ m	Pore size
rho_fluid	1000[kg/m^3]	1000 kg/m³	Fluid density
eta_fluid	1e-3[Pa*s]	0.001 Pa·s	Fluid viscosity
gamma_surface	0.072[N/m]	0.072 N/m	Surface tension
P_cap	2*gamma_surface/pore_size	7200 N/m²	Capillary pressure
C0_mol	7e-8[mol/m^3]	7 × 10⁻⁸ mol/m³	Nanochain concentration
D0	2e-13[m^2/s]	2 × 10⁻¹³ m²/s	Nanochain diffusion coefficient
delta_smooth	100e-6[m]	1 × 10⁻⁴ m	Wetting front smoothing width
power_contrast	2	2	Contrast power for effective properties
P_inlet	25,000 [Pa]	25,000 Pa	Inlet pressure
k_min	0.01	0.01	Minimum relative permeability
W_TL	1 [mm]	0.001 m	Test line width
H_TL	H_membrane	0.0025 m	Test line height
x_testline	L_model	0.003 m	Test line position
w_testline	W_TL	0.001 m	Test line width
C_Ab_init	1e-5[mol/m^3]	1 × 10⁻⁵ mol/m³	Initial antibody concentration at test line
kon	N_Abs*1e4[1/(M*s)]	1000 m³/(s·mol)	Association rate constant
koff	1e-9[1/s]	1 × 10⁻⁹ s⁻¹	Dissociation rate constant
N_Abs	100	100	Binding efficiency parameter (sweep variable)
k_recruit	1*7e7[1/(M*s)]	70,000 m³/(s·mol)	Magnetic recruitment constant (sweep variable)
V_chain	2e-20[m^3]	2 × 10⁻²⁰ m³	Nanochain volume
c3_max	70e-6[mol/m^3]	7 × 10⁻⁵ mol/m³	Maximum surface binding capacity

Step 3: Define variables for wetting front and effective properties

• Navigate to Definitions → Variables
• Create "Variables 1″ (Geometric Entity Selection: Entire model): Table 6

**Table 6:** Variables for wetting front and transport properties.

**Name**	**Expression**	**Unit**	**Description**
x_front	sqrt(2*k_perm*P_cap*(*t* + 0.1)/(eta_fluid*porosity))	m	Wetting front position (Lucas-Washburn)
wetness	0.5*(1 + tanh((x_front - x)/delta_smooth))	-	Smooth wetting indicator (0→1)
k_eff	k_perm * (k_min + (1-k_min)*wetness^power_contrast)	m²/s	Effective permeability
D_eff	D0 * max(wetness, 0.01)	m²/s	Effective diffusion coefficient
on_testline	(≥ *x*_testline)*(≤ *x*_testline + *w*_testline)	-	Test line indicator

Step 4: Build geometry

Navigate to Geometry 1

• Create membrane domains:
• BeforeTL (region before test line):
  Right-click Geometry 1 → Rectangle
  Label: BeforeTL
  Width: L_model
  Height: H_membrane
  Position, Base: Corner
  x: 0
  y: 0
  Click Build Selected
• TL (test line region):
  Right-click Geometry 1 → Rectangle
  Label: TL
  Width: W_TL
  Height: H_TL
  Position, Base: Corner
  x: L_model
  y: 0
  Click Build Selected
• AfterTL (region after test line):
  Right-click Geometry 1 → Rectangle
  Label: AfterTL
  Width: L_model
  Height: H_membrane
  Position, Base: Corner
  x: L_model+*W*_TL
  y: 0
  Click Build Selected
• Form Union:
  Right-click Geometry 1 → Form Union
  Click Build All Objects

Step 5: Configure Darcy's Law physics (dl)

• Navigate to Darcy's Law (dl)
• Click Fluid 1
  Domain Selection: All domains
  Density (ρ): rho_fluid
  Dynamic viscosity (μ): eta_fluid
• Click Porous Matrix 1
  Domain Selection: All domains
  Porosity (ε_p): porosity
  Permeability model: Permeability
  Permeability (κ): k_eff (user defined, isotropic)
• Right-click Darcy's Law → No Flow
  Label: No Flow 1
  Boundary Selection: All boundaries (will override specific boundaries next)
• Right-click Darcy's Law → Inlet
  Label: Inlet 1
  Boundary Selection: Manual → Select boundary 1 (left edge, *x* = 0)
  Boundary condition: Pressure
  Pressure (p₀): P_inlet
• Right-click Darcy's Law → Outlet
  Label: Outlet 1
  Boundary Selection: Manual → Select boundary 10 (right edge)
  Boundary condition: Pressure
  Pressure (p₀): 0
• Click Initial Values 1
  Domain Selection: All domains
  Pressure: 0

Step 6: Configure Transport of Diluted Species - Nanochains (tds)

• Navigate to Transport of Diluted Species (tds)
  Label: tds_nanochains
  Name: tds
• Click Transport Properties 1
  Domain Selection: All domains
  Convection:
    ▪ Velocity field: Darcy's velocity field (dl/porous1)
  Diffusion:
    ▪ Diffusion coefficient (D_c): Material (will define in material)
  Additional transport mechanisms:
    ▪ Mass transfer in porous media
    ▪ Out-of-Plane Thickness (d_z): 3[um]
• Click No Flux 1
  Boundary Selection: All boundaries
• Right-click tds → Inflow
  Label: Inflow 1
  Boundary Selection: Manual → Select boundary 1
  Concentration (c₀,c): C0_mol
  Boundary condition type: Concentration constraint
• Right-click tds → Outflow
  Label: Outflow 1
  Boundary Selection: Manual → Select boundary 10
• Right-click tds → Reactions
  Label: Reactions 1
  Domain Selection: Manual → Select domains 1, 2, 3 (all regions)
  Reaction Rates:
    ▪ R_c (nanochain consumption): -kon*c*c2 + koff*c3 - k_recruit*c*c3
• Click Initial Values 1
  Domain Selection: All domains
  Concentration (c): 0

Step 7: Configure Transport of Diluted Species - Antibody/Complexes (tds2)

• Settings:
  Label: tds_Abs_and_complexes
  Name: tds2
• Click Transport Properties 1
  Domain Selection: All domains
  Convection: ✗ (unchecked) – antibodies are immobilized at test line
  Diffusion: Off (antibodies don't diffuse, bound to membrane)
• Click No Flux 1
  Boundary Selection: All boundaries
• Right-click tds2 → Reactions
  Label: Reactions 1
  Domain Selection: Manual → Select domains 1, 2, 3
  Reaction Rates:
    ▪ R_c2 (free antibody consumption): -kon*c*c2 + koff*c3
    ▪ R_c3 (complex formation): kon*c*c2 – koff*c3 + k_recruit*c*c3
• Right-click tds2 → Initial Values
  Label: Initial Values 1
  Domain Selection: All domains
  Concentration:
    ▪ c2 (antibody): C_Ab_init * on_testline (only at test line initially)
    ▪ c3 (complex): 0

Step 8: Generate mesh

Navigate to Mesh 1

• Click Size
  Geometric entity level: Entire geometry
  Element size: Predefined → Finer
• Click Build All

Step 9: Configure study (Parametric Sweep ± Time Dependent)

Navigate to Study 1

Add Parametric Sweep:

• Right-click Study 1 → Parametric Sweep
• Move Parametric Sweep node above "Step 1: Stationary"
• Click Parametric Sweep
  Sweep type: All combinations
  Parameter names: N_Abs, k_recruit
  Parameter value list:
    ▪ N_Abs: 300,400,500,600,700,800,900,1000 (binding efficiency variations)
    ▪ k_recruit: 0,70,000 (cooperative recruitment: off vs on)
  Parameter units:
    ▪ N_Abs: (dimensionless)
    ▪ k_recruit: m^3/(s*mol)
  Keep solutions: All

Step 1: Stationary

• Click Step 1: Stationary
  Physics and Variables Selection:
    ▪ Solve for: Darcy's Law (dl) only (compute steady-state flow field first)

Step 2: Time Dependent

• Click Step 2: Time Dependent
  Times: range(0,1360) (0 to 360 s with 1 s output interval)
  Physics and Variables Selection:
    ▪ Solve for: All (tds_nanochains, tds_Abs_and_complexes)
  Tolerance: Physics controlled

Step 10: Run simulation

• Click Compute button (=) in toolbar
• Simulation will run: 8 N_Abs values × 2 k_recruit values = 16 parameter combinations
• Monitor progress in Progress window

## SEM sample preparation for membrane integrity assessment

To exclude potential mechanical damage from rotating magnetic fields, we performed scanning electron microscopy of nitrocellulose membranes before and after magnetic rotation. Three sample groups were prepared: (i) unused control membranes, (ii) membranes used in standard lateral flow assays without magnetic field, and (iii) membranes exposed to magnetic rotation (3 Hz, 200 Oe) during 6-minute lateral flow assays.

After assay completion, membrane samples (5 mm × 5 mm) were air-dried for 24 h at room temperature. For SEM imaging, samples were sputter-coated with 5 nm gold film (99.9 % purity, Girmet, Russia) using cold magnetron sputtering in argon atmosphere (DSCR Desktop Sputter/Carbon Coater, NanoStructured Coatings Co., Iran). Coating parameters: chamber pressure 50 mTorr, target current 10 mA, deposition time approximately 15 min.

Samples were imaged using a Tescan MAIA3 field-emission SEM at 20 kV accelerating voltage, working distance 4.99 mm, and magnification 3270 × (field of view 63.5 µm). The in-beam secondary-electron detector was used to maximize surface topography contrast.

## Implementation considerations and inter-laboratory variability

The workflows described here were designed with replicability in mind, but require access to several specialized tools. The COMSOL Multiphysics simulations require a commercial license (academic licenses are widely available through institutional subscriptions); the underlying physics formulations — Navier-Stokes equations with rotating wall boundary conditions, and Darcy flow coupled with reaction-diffusion — are standard and can also be implemented in open-source CFD frameworks, at the cost of increased setup effort. SEM characterization can be complemented or substituted with transmission electron microscopy; dynamic light scattering provides a bulk hydrodynamic size distribution but does not resolve the anisotropic morphology of nanochains and is therefore of limited utility for this system. The rotating magnetic field generator is assembled from standard commercial components (NdFeB permanent magnets, DC gear-motor, aluminium frame) and does not require specialized fabrication. Detection is not restricted to a specific modality: the signal enhancement produced by rotating nanochains manifests as increased test-line intensity observable even by direct visual inspection and standard optical readouts, so laboratories can implement the approach using smartphone imaging, flatbed scanning, MPQ, or any other quantitative test-line readout compatible with their existing workflow.

Principal sources of inter-laboratory variability are expected to originate from (i) nanochain batch-to-batch reproducibility, which is mitigated by the standardized synthesis protocol described in the companion article [1], and (ii) rotating field amplitude and homogeneity, which we characterized in the present work (below 3 % variation across the central zone, see the "Rotating magnetic field generator" subsection).

## Method validation

### Validation of antigen capture efficiency measurement procedure

The antigen capture efficiency measurement procedure was validated by comparing rotating versus static incubation conditions. Using 1 ng/mL H-FABP as model analyte, the method demonstrated clear differentiation between conditions: 52 ± 11 % capture under rotation versus 16 ± 10 % without rotation (*n* = 3, Table S1). The control samples without nanochains showed 95 ± 8 % recovery, confirming minimal nonspecific losses. These results validate both the measurement procedure and the enhancement effect of magnetic rotation.

### Computational fluid dynamics model validation

**Fig. 1:**
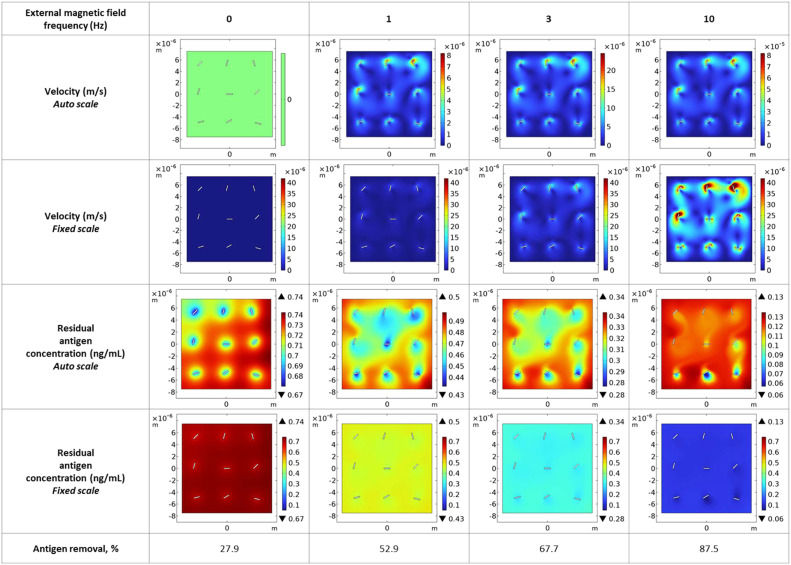
Computational fluid dynamics simulation results demonstrating convective enhancement by rotating magnetic nanochains. Velocity magnitude fields (rows 1–2) and residual antigen concentration distributions (rows 3–4) at steady state (*t* = 360 s) for rotation frequencies of 0, 1, 3, and 10 Hz. Both auto-scaled (rows 1, 3) and fixed-scale (rows 2, 4) visualizations are shown for comparison. Antigen removal percentages (bottom row) demonstrate progressive enhancement with increasing rotation frequency, from 27.9 % under static conditions to 87.5 % at 10 Hz. At the experimentally optimized frequency of 3 Hz, the model predicts 67.7 % antigen removal, representing improvement over static conditions. Domain size: 15 µm × 15 µm containing nine 1.05 µm × 0.15 µm nanochains.

The computational fluid dynamics model was validated by comparing simulation predictions with experimental measurements of antigen capture efficiency under rotating and static conditions. Fig. 1 presents comprehensive visualization of the simulation results across four rotation frequencies (0, 1, 3, and 10 Hz). The figure displays velocity fields (rows 1–2) and residual antigen concentration distributions (rows 3–4) at steady state (*t* = 360 s), shown with both auto-scaled and fixed color scales for comparison.

At static conditions (0 Hz), fluid velocity remains negligible throughout the domain (<10⁻⁸ m/s, shown in green/dark blue). Upon activation of rotation, localized velocity patterns emerge around each nanochain. At 1 Hz, velocity magnitudes reach approximately 5 × 10⁻⁶ m/s in regions immediately adjacent to rotating nanochains. At the experimentally optimized frequency of 3 Hz, maximum velocities of ∼20 × 10⁻⁶ m/s are observed, generating distinct vortex patterns that extend several micrometers from each nanochain surface. At 10 Hz, velocity fields intensify further to ∼40 × 10⁻⁶ m/s, creating strong convective mixing throughout the entire domain. The fixed-scale visualization (row 2) clearly demonstrates the progressive enhancement of fluid motion with increasing rotation frequency.

The residual antigen concentration maps reveal the impact of convective enhancement on binding kinetics. Under static conditions (0 Hz), narrow depletion zones form only immediately adjacent to nanochain surfaces (green regions in high-concentration red background), indicating diffusion-limited transport. The bulk concentration remains near initial values (0.74 ng/mL = 74 % of 1.0 ng/mL starting concentration), corresponding to only 27.9 % antigen removal. At 1 Hz, depletion zones expand modestly, and overall concentration decreases (yellow regions), achieving 52.9 % removal. At 3 Hz, the depletion zones become substantially more extended, and the domain-average concentration drops significantly (cyan/light blue), reaching 67.7 % removal—a 2.4-fold improvement over static conditions. At 10 Hz, the concentration field becomes nearly uniformly depleted (dark blue), indicating rapid convective transport and binding across the entire domain, with 87.5 % antigen removal.

The simulation captures the correct mechanistic trend: rotation substantially enhances antigen binding. A direct numerical comparison between model predictions and experimental measurements at static and optimal rotation conditions is presented in Table 7.

**Table 7:** Direct comparison between CFD model predictions and experimental measurements of antigen capture.

**Frequency (Hz)**	**Predicted removal (%)**	**Experimental capture (%)**
0 (static)	27.9	16 ± 10
3 (optimum)	67.7	52 ± 11

The CFD model and the experimental capture measurement operate in different configurations: the model simulates an idealized 2D domain with nine nanochains in free solution, whereas the experimental values were obtained by supernatant depletion after incubation of nanochains with antigen in buffer. Despite these differences in geometry and boundary conditions, both approaches consistently demonstrate the same effect: nanochain rotation at 3 Hz enhances antigen capture approximately 2–3-fold compared to static conditions (predicted ratio 2.4, experimental ratio 3.3). The model thus correctly identifies the physical mechanism — convective enhancement of mass transport by rotating nanochains — and provides reliable guidance for frequency optimization within the validated 0–3 Hz range.

The CFD model is validated in the low-frequency regime (0–3 Hz), where nanochains maintain synchronous rotation with the external field. In this regime, the predicted enhancement of binding efficiency with frequency (27.9 % at 0 Hz to 67.7 % at 3 Hz) is consistent with the experimentally observed trend. Three physical mechanisms, not incorporated in the current model, are responsible for the divergence above 3 Hz: (i) loss of synchronous rotation — beyond a critical frequency, viscous drag torque exceeds magnetic driving torque, and nanochains transition from phase-locked rotation to oscillatory or slip-stick motion, reducing effective stirring; the critical frequency depends on chain geometry, field amplitude, and medium viscosity, and lies within the experimentally explored range for our system; (ii) insufficient contact time – at 10 Hz, each binding site completes rotation in 100 ms, which may be inadequate for encounter complexes to form stable immunocomplexes; (iii) shear-induced destabilization of forming immunocomplexes at elevated rotational velocities.

Accordingly, the quantitative agreement between model and experiment is rigorously established within the 0–3 Hz range that corresponds to the experimentally identified optimum; extension to higher frequencies would require explicit modeling of torque balance, chain flexibility, and frequency-dependent binding kinetics.

### Computational nanochain transport dynamics validation

**Fig. 2:**
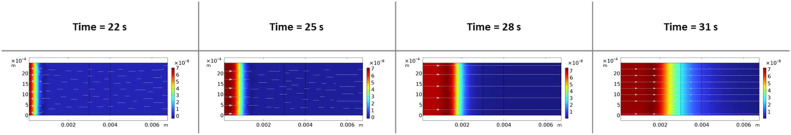
Computational temporal evolution of nanochain concentration during early-stage membrane migration at four time points (22, 25, 28, and 31 s after sample application).

Fig. 2 presents the temporal evolution of nanochain concentration (c) during early-stage membrane migration at four time points: 22, 25, 28, and 31 s after sample application. These snapshots visualize the advancing wetting front and nanochain distribution before reaching the test line region (located at *x* = 3 mm). At *t* = 22 s, the nanochain-containing front has just entered the computational domain, with concentration confined to the leftmost region. By *t* = 31 s, the front has advanced approximately halfway through the membrane. Notably, a subtle but reproducible concentration gradient is visible within the wetted region: nanochain density increases slightly toward the leading edge of the wetting front (visible as the yellow-orange gradient ahead of the main blue region). This accumulation arises from convective transport outpacing diffusive relaxation at the moving boundary, creating a "compression" zone where nanochains are swept forward by capillary flow faster than they can redistribute by diffusion. Critically, this early-stage transport pattern is independent of binding parameters (N_Abs and k_recruit) because nanochains have not yet reached the test line where antibody-antigen interactions occur. The slight front-edge enrichment observed here has mechanistic significance: when this concentrated leading edge first encounters the test line antibodies, it initiates the earliest binding events, potentially seeding the spatial asymmetry that subsequently develops under cooperative capture conditions.

**Fig. 3:**
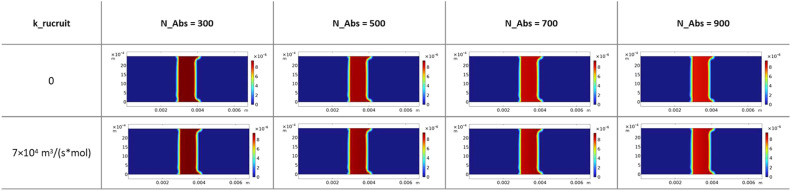
Computational spatial distribution of free antibodies at test line after 360 s.

Fig. 3 displays the spatial distribution of free antibodies (c2) at the test line region (*x* = 3–4 mm) at the end of the simulation (*t* = 360 s), comparing eight N_Abs values ranging from 300 to 1000 under two conditions: k_recruit = 0 representing standard binding, and k_recruit = 7 × 10⁴ m³/(s·mol) representing cooperative binding. Both auto-scaled and fixed-scale representations are shown to facilitate quantitative comparison. Even at the highest binding efficiency (N_Abs = 1000), the free antibody concentration remains above 5 × 10⁻⁶ mol/m³, representing approximately 50 % of the initial antibody concentration (C_Ab_init = 10⁻⁵ mol/m³). This confirms that the model operates in the non-saturating regime where antibody availability does not limit capture, a design choice that isolates the effect of cooperative recruitment from depletion artifacts. Under cooperative binding conditions (k_recruit = 7 × 10⁴), particularly visible at intermediate N_Abs values between 500 and 700, antibody depletion becomes more pronounced at the upstream edge (*x* = 3.0–3.2 mm) compared to downstream regions (*x* = 3.8–4.0 mm). This spatial pattern reflects preferential complex formation at the front edge, consistent with the cooperative capture mechanism. In contrast, standard binding conditions (k_recruit = 0) produce nearly uniform antibody depletion across the test line width at all N_Abs values, as expected for diffusion-limited binding without spatial coupling. This analysis validates that the simulation parameters were chosen to represent the experimental conditions where the test line operates below saturation, matching the regime where 5-fold signal amplification was observed experimentally (see companion article in Biosensors and Bioelectronics journal). If antibody saturation occurred, cooperative effects would be masked by complete depletion, obscuring the fundamental mechanism under investigation.

**Fig. 4:**
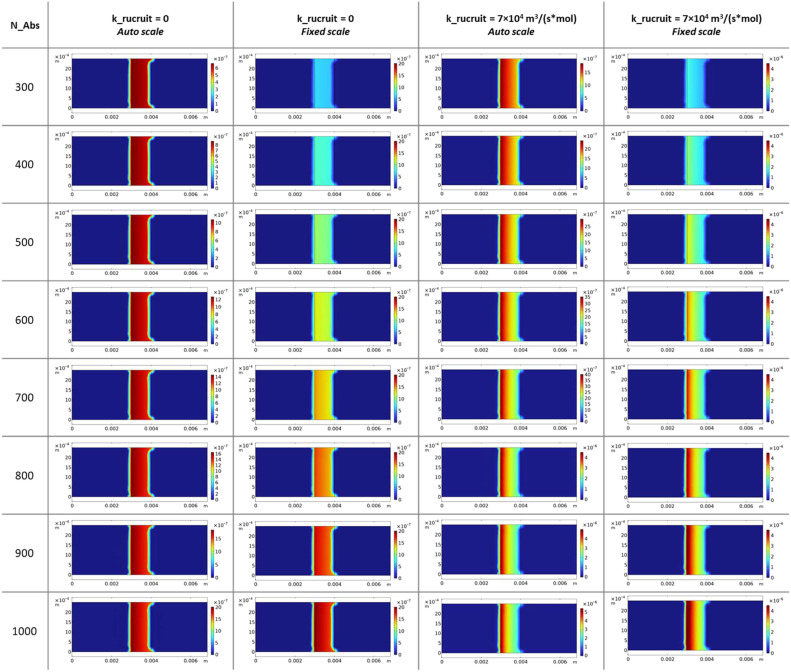
Computational complex formation patterns under standard and cooperative binding conditions.

Fig. 4 presents the spatial distribution of antibody-nanochain complexes (c3) at the test line at *t* = 360 s, directly addressing the central hypothesis of cooperative capture. The figure compares standard binding (k_recruit = 0) versus cooperative binding (k_recruit = 7 × 10⁴ m³/(s·mol)) across eight N_Abs values, with both auto-scaled and fixed-scale visualizations to reveal subtle spatial features. Without cooperative recruitment (k_recruit = 0), complex distribution remains uniformly distributed across the test line width (*x* = 3.0–4.0 mm) at all N_Abs values. Maximum complex density increases with N_Abs, reflecting higher binding efficiency. The fixed-scale visualization confirms homogeneous capture throughout the test line region, consistent with standard Langmuir-type binding where capture probability depends only on local concentrations of free nanochains and antibodies. When cooperative recruitment is activated (k_recruit = 7 × 10⁴), dramatic spatial asymmetry develops. Maximum complex density shifts decisively toward the upstream edge (*x* ≈ 3.0–3.3 mm), with the asymmetry intensifying as N_Abs increases. This behavior quantitatively reproduces the experimental observation reported in companion article in Biosensors and Bioelectronics journal. Critically, this gradient develops even under non-saturating conditions confirmed in Fig. 3, establishing that asymmetry arises from enhanced local capture rather than antibody depletion artifacts.

The physical mechanism underlying this divergence can be understood through the interplay of transport and binding kinetics. The standard binding model predicts uniform capture because nanochain concentration arriving at the test line becomes spatially uniform after diffusive equilibration following front passage, free antibody availability remains uniform in the non-saturating regime, and the capture rate proportional to kon·c·c2 therefore exhibits no spatial variation. The cooperative binding model introduces positive feedback through the magnetic recruitment term k_recruit·c·c3. The slightly enriched nanochain front visible in Fig. 2 reaches the upstream edge first, forming initial complexes. These accumulated complexes then magnetically attract flowing nanochains, locally enhancing capture probability. Enhanced upstream capture depletes the flowing nanochain population, reducing the concentration available for downstream regions. Each subsequently captured nanochain further enhances the local capture rate, creating a self-reinforcing spatial gradient that amplifies over time. Quantitative agreement between simulation and experiment validates the proposed mechanism.

The computational model demonstrates that standard Langmuir-type binding cannot reproduce the experimentally observed spatial patterns, as uniform antibody and nanochain distributions necessarily lead to uniform capture. Cooperative capture quantitatively reproduces experimental asymmetry through the k_recruit term representing magnetic attraction between flowing and immobilized nanochains, generating concentration-dependent spatial gradients that match experimental observations. The mechanism operates robustly in non-saturating conditions where antibody availability is non-limiting, confirming that cooperation rather than depletion drives the observed pattern.

### SEM validation of membrane structural integrity

**Fig. 5:**
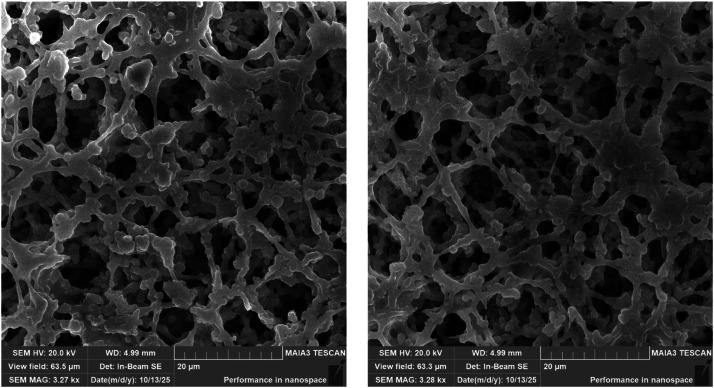
Scanning electron microscopy of nitrocellulose membrane fibrous structure: without magnetic field (left) and after magnetic rotation at 3 Hz, 200 Oe (right).

Representative SEM images of nitrocellulose membrane before and after magnetic rotation are shown in Fig. 5. Both control and magnetically-rotated membranes exhibited the characteristic porous fibrous structure of nitrocellulose NC140 grade: fiber diameters of 0.3–1.5 µm, pore sizes of 5–25 µm, and an interconnected three-dimensional network consistent with manufacturer specifications (Sartorius Unisart NC140).

Qualitative comparison revealed no detectable differences in fiber integrity, pore structure, or overall membrane architecture between unused controls and membranes exposed to magnetic rotation. Specifically, no fiber breakage, fragmentation, pore collapse, densification, surface roughening, deformation, or embedded nanochain aggregates disrupting the fibrous network were observed. The smooth fiber surfaces and intact pore structure remained identical across all conditions. These observations confirm that magnetic forces acting on rotating nanochains, estimated at piconewton scale from magnetic dipole calculations, are insufficient to mechanically disrupt the cellulose fiber network. The preserved membrane architecture validates the mechanical compatibility of magnetic actuation with standard lateral flow platforms and excludes structural artifacts as contributors to observed signal enhancements.

### Parameter sensitivity and experimental uncertainty

The parametric sweep across N_Abs (300–1000) and k_recruit (0 and 7 × 10⁴ m³/(s·mol)), described in Section "Computational Modeling of Nanochain Transport and Cooperative Binding in Porous Lateral Flow Membrane", provides quantitative insight into model sensitivity. Predicted complex density at the test line varies approximately 3.5-fold across the N_Abs range, indicating high sensitivity to binding efficiency parameters. Activating cooperative recruitment (k_recruit > 0) primarily affects the spatial distribution of captured nanochains along the test line, with the total complex amount changing by <15 %, indicating that the cooperative mechanism manifests predominantly as spatial redistribution of capture events rather than as bulk amplification of binding.

On the experimental side, the principal sources of measurement uncertainty for the methods described here are: nanochain concentration (controlled by MPQ-based mass calibration), antibody loading density (quantified by BCA assay), and rotating field amplitude (verified by Hall magnetometry and characterized for spatial homogeneity, as described in the "Rotating magnetic field generator" subsection). The combined effect of these contributors is reflected in the inter-assay coefficient of variation below 10 % across the dynamic range, as reported in the companion article [1].

### Limitations

The supernatant depletion method requires magnetic separation and assumes negligible analyte loss during handling. It is applicable only to magnetic labels and may underestimate capture efficiency if nanochain aggregation occurs during separation.

The CFD model employs a two-dimensional domain, which increases the effective surface-to-volume ratio compared to three-dimensional geometry and may contribute to overestimation of local binding rates. The rigid-body assumption neglects nanochain flexibility and polydispersity; TEM analysis reported in the companion article [1] reveals a mean aspect ratio of 4.7 ± 1.5, lower than the mean particle count of 8 ± 2 per chain, indicating bending and kinking that would modify the flow field generated during rotation. Hydrodynamic interactions between nanochains are not modeled; at the experimental concentration used for capture efficiency measurements (3 μg in 50 μL), the mean inter-particle distance estimated from the chain dimensions (1.05 μm × 0.15 μm) and nanochain mass density is of the order of 10 μm, approximately an order of magnitude larger than the chain length itself. Under these conditions, inter-chain hydrodynamic coupling is expected to be a secondary effect relative to the single-chain convective mechanism captured by the model. Quantitative extension to concentrated regimes, where multi-body hydrodynamic interactions become significant, represents an important direction for further development.

The Darcy flow model for cooperative binding simplifies the three-dimensional porous membrane as a 2D domain with effective permeability. Cooperative recruitment is represented by a phenomenological rate constant (k_recruit) rather than explicit magnetic dipole-dipole interactions. The wetting front is modeled analytically (Lucas-Washburn) rather than solving full two-phase flow, which may not capture transient flow instabilities.

## Declaration of competing interest

The authors declare that they have no known competing financial interests or personal relationships that could have appeared to influence the work reported in this paper.

## Data Availability

Data will be made available on request.

## Appendix Supplementary materials

Image, application 1
