# Supplementary material for: Computational modeling and characterization methods for rotating magnetic nanochain-enhanced lateral flow immunoassays
Source: MethodsX. 2026 May 5;16:103936. doi: 10.1016/j.mex.2026.103936 (PMC13200043; doi:10.1016/j.mex.2026.103936)
Supplement: Supplementary file 1 [file mmc1.docx]

***Supplementary material***

**Computational modeling and characterization methods for rotating magnetic nanochain-enhanced lateral flow Immunoassays**

*Alexey V. Orlov^1^, Juri A. Malkerov^1,2^, Alexandra S. Rakitina^1,3^, Anastasiia Kudriavtseva^1,3^, Alexander A. Minakov^1^, Daniil I. Tselikov^2^, Petr I. Nikitin^1,2,*^, Slavko Kralj^4,5,*^*

^1^ Prokhorov General Physics Institute of the Russian Academy of Sciences, Moscow, Russia

^2^ National Research Nuclear University MEPhI (Moscow Engineering Physics Institute), 31 Kashirskoe shosse, 115409, Moscow, Russia

^3^ Moscow Center for Advanced Studies, 20 Kulakova Str., Moscow, Russia

^4^ Jožef Stefan Institute, Department for Materials Synthesis, Jamova cesta 39, SI-1000 Ljubljana, Slovenia, Europe

^5^ University of Ljubljana, Faculty of Pharmacy, Aškerčeva 7, SI-1000 Ljubljana, Slovenia, Europe

**Corresponding author: nikitin@kapella.gpi.ru (P.I.N.), slavko.kralj@ijs.si (S.K.)*

**Supplementary Note 1. Direct measurement of antigen capture efficiency**

To provide direct evidence for enhanced antigen binding under magnetic rotation, we performed supernatant analysis after incubating magnetic nanochains with H-FABP samples under rotating and static conditions (Table S1).

**Table S1.** Residual H-FABP concentration in supernatants after nanochain incubation

| **Condition** | **Residual H-FABP (ng/mL)** | **Capture efficiency (%)** | **n** |
| --- | --- | --- | --- |
| Control (no nanochains) | 0.95 ± 0.08 | - | 3 |
| Static incubation | 0.84 ± 0.10 | 16% | 3 |
| Rotation (3 Hz, 200 Oe) | 0.48 ± 0.11 | 52% | 3 |

Capture efficiency was calculated as: *(Initial concentration - Residual concentration) / Initial concentration × 100%.* The improvement in capture efficiency under rotation directly demonstrates enhanced antibody-antigen binding kinetics.
